# The Association Between miRNA-223-3p Levels and Pain Severity in Fibromyalgia Syndrome: A Molecular Approach

**DOI:** 10.3390/ijms27010176

**Published:** 2025-12-23

**Authors:** Zerrin Barut, Özlem Karataş, Fatma Tuba Akdeniz, Bürke Çırçırlı, Serpil Demir, Turgay İsbir

**Affiliations:** 1 Department of Basic Medical Sciences, Faculty of Dentistry, Antalya Bilim University, 07910 Antalya, Türkiye; 2 Department of Internal Medicine, Division of Physical Medicine and Rehabilitation, Faculty of Medicine, Akdeniz University, 07070 Antalya, Türkiye; drozlemkaratas86@gmail.com (Ö.K.); serpiltuna@akdeniz.edu.tr (S.D.); 3 Department of Genetics and Bioengineering, Faculty of Engineering and Naturel Sciences, Istanbul Okan University, 34959 Istanbul, Türkiye; fatma.akdeniz@okan.edu.tr; 4 Department of Medical Biotechnology and Medical Biochemistry, Faculty of Medicine, Akdeniz University, 07070 Antalya, Türkiye; burkecircirli@outlook.com; 5 Institute of Health Sciences, Department of Molecular Medicine, Yeditepe University, 34755 Istanbul, Türkiye; turgay.isbir@yeditepe.edu.tr

**Keywords:** fibromyalgia syndrome (FMS), miRNA-223-3p, neuroinflammation, pain severity, Visual Analog Scale, biomarker, chronic pain

## Abstract

Fibromyalgia Syndrome (FMS) is a chronic syndrome commonly characterized by widespread musculoskeletal pain and fatigue. Current evidence suggests that FMS diagnosis relies on clinical evaluation and patient-reported symptoms. MicroRNAs, which serve as key regulators of gene expression, have been proposed to play a role in the pathogenesis of FMS and other chronic pain syndromes. In this pilot study, miRNA-223-3p expression levels were examined in patients with FMS, and their relationship with pain intensity—assessed using the Visual Analog Scale (VAS)was evaluated. To obtain a broader understanding of the inflammatory response, serum interleukin-1 beta (IL-1β) levels were also measured. miRNA-223-3p expression levels were significantly reduced in the FMS group compared with healthy controls (*p* < 0.05), whereas IL-1β levels did not differ significantly between the groups (*p* = 0.1135). The negative correlation between miRNA-223-3p and VAS scores indicates that lower miRNA levels are associated with increased pain severity. Overall, these results suggest that reduced miRNA-223-3p expression levels may be linked to neuroimmune processes and heightened pain perception in FMS. The findings provide valuable preliminary insights that may guide future studies with larger sample sizes.

## 1. Introduction

Fibromyalgia syndrome (FMS) is a chronic disorder characterized by persistent and widespread musculoskeletal pain, most commonly affecting women, and is frequently accompanied by sleep disturbances, fatigue, cognitive impairment, and a variety of somatic symptoms [1,2,3]. Its etiology is multifactorial, involving the interplay of psychological, biochemical, and immunological mechanisms, as well as oxidative stress and environmental triggers. Neuroendocrine dysfunction and genetic susceptibility are also believed to play critical roles in its pathogenesis [4,5,6,7].

Fibromyalgia is characterized by exaggerated responses to both nociceptive and non-nociceptive stimuli and is considered a manifestation of central pain processing dysfunction; this enhanced central sensitization is associated with hyperalgesia and allodynia arising from aberrant peripheral nociceptive input [8,9]. Although pain serves as a protective physiological mechanism, its chronicity can lead to substantial reductions in quality of life and impose significant functional limitations. Current therapeutic strategies often remain insufficient; the limited efficacy of conventional analgesics and their potential for adverse effects with long-term use further complicate chronic pain management in fibromyalgia, underscoring the need for more effective and targeted treatment approaches [10,11].

The diagnostic criteria proposed by the American College of Rheumatology (ACR) in 2016 incorporate various pain indices, including the Visual Analog Scale (VAS). The VAS is widely utilized in both clinical practice and research settings as a highly valid and reliable method for assessing the severity of chronic pain [12,13,14]. Despite the availability of these diagnostic tools, it is estimated that nearly 75% of individuals with FMS remain undiagnosed [15]. In recent years, growing evidence has highlighted the role of epigenetic mechanisms—through which environmental factors exert long-term effects on gene expression—in the development and persistence of chronic pain [16]. In this context, a range of molecular biomarkers, including microRNAs (miRNAs), has been proposed for their potential contributions to the pathophysiology and objective assessment of FMS [17,18].

MiRNAs are small non-coding RNA molecules that regulate gene expression at the post-transcriptional level and exhibit high stability in cells and body fluids [19,20]. They can modulate immune responses, contribute to the development and persistence of pain syndromes, and display distinct expression patterns depending on the etiology of chronic pain [21]. Circulating miRNAs have been investigated as potential biomarkers in various neurological disorders [22]. Their ability to regulate genes and proteins involved in pain pathways suggests that these molecules may hold value not only diagnostically but also therapeutically [23,24]. Furthermore, miRNAs have the capacity to modulate reactive oxygen species (ROS) and are themselves influenced by oxidative stress, highlighting their bidirectional role in pathophysiological processes [25,26]. Taken together, these interactions may suggest that miRNAs may represent promising diagnostic and therapeutic targets in FMS.

miRNA-223-3p is a hematopoietic-specific microRNA that plays a critical role in myeloid cell development [27]. It modulates neutrophil and macrophage activity, suppresses pro-inflammatory responses, and may reduce neuronal excitability by influencing central pain processing [28,29]. Through the regulation of the NF-κB pathway, miRNA-223-3p can further modulate immune responses [22]. By binding to the 3′-UTR of *NLRP3* (NOD-like receptor protein 3), miRNA-223-3p suppresses inflammasome activation and reduces inflammation [30]. Similarly, studies in liver injury models have reported that synthetic miRNA-223-3p analogs attenuate inflammation by downregulating *NLRP3* expression [31]. Moreover, miRNA-223-3p has been found to target the transcription factors FOXO1 and FOXO3a, implicating it in critical processes such as cellular stress responses, autophagy, immune regulation, and tissue repair [32]. These multifaceted molecular actions extend beyond inflammation and play notable roles across diverse pathological contexts. For instance, in cancer models, miRNA-223-3p has been shown to promote cell proliferation and invasion by targeting *FBXW7* (F-box and WD repeat domain-containing 7), while also influencing intercellular communication and tissue responses by suppressing *SORBS1* (Sorbin and SH3 domain-containing protein 1) through microvesicles released from cancer-associated fibroblasts [33,34]. Experimental neuroinflammation models provide complementary findings. Overexpression of miRNA-223-3p in microglial cells significantly reduced M1 polarization markers—including iNOS, COX-2 (Cyclooxygenase-2), TLR4, NLRP3, and NF-κB—alongside decreases in IL-1β, IL-6, TNF-α, and oxidative stress indicators [35].

In addition, miRNA-223-3p has been shown to exert analgesic effects by inhibiting the MAPK pathway via targeting *MKNK2* (MAPK-interacting serine/threonine-protein kinase 2), and in the adult brain it displays neuroprotective properties by regulating synaptic function and glutamate receptor signaling [5,36,37]. It has also been reported to attenuate IL-8–induced injury in hippocampal neurons and to alleviate anxiety- and depression-like behaviors associated with the chronic unpredictable mild stress (CUMS) model [38]. Since these proteins regulated by miRNA-223-3p are considered fundamental components of tissue repair mechanisms that influence chronic pain and inflammatory processes, our study focused on miRNA-223-3p.

Clinical studies have demonstrated that elevated circulating levels of pro-inflammatory cytokines in FM are associated with immune dysregulation, while alterations in pain-related neurotransmitters contribute to neural dysfunction. Significant changes in pro-inflammatory cytokine levels have been reported in both the serum and tissue biopsies of FM patients [39,40]. Given that FMS has been reported to be associated with an increased pro-inflammatory cytokine burden and considering the involvement of miRNA-223-3p in molecular networks regulating inflammatory responses, IL-1β was included in this study as a component of the miRNA-223-3p–modulated inflammatory axis.

In patients with FMS, findings suggestive of inflammatory processes have been reported; in this context, IL-1β is characterized by pro-inflammatory activity across multiple tissues, and its effects on nociceptors may amplify pain transmission and perception via various ion channels. Increased IL-1β activity has been linked to numerous autoimmune disorders in which pain is also a common accompanying symptom, including inflammatory bowel disease, gout, multiple sclerosis, and rheumatoid arthritis [41,42,43,44].

Several studies have reported that, in various diseases, miRNA-223-3p plays a regulatory role in IL-1β expression by suppressing caspase-1 and NLRP3 inflammasome activity, thereby reducing IL-1β production and limiting the intensity of the inflammatory response [42,45,46]. This interaction forms a protective mechanism that prevents excessive activation of inflammatory pathways, consequently reducing tissue damage and inflammation severity. Thus, the miRNA-223-3p/NLRP3/IL-1β axis is considered a critical pathway in the molecular regulation of inflammation [47,48,49].

Despite the growing recognition of microRNAs in chronic pain mechanisms, no study to date has directly examined the relationship between serum miRNA-223-3p and IL-1β levels and pain severity (VAS scores) in patients with fibromyalgia. Existing evidence on the role of miRNA-223-3p in regulating IL-1β production suggests that investigating this molecular interaction within the context of fibromyalgia may provide valuable insights into the mechanisms underlying chronic pain and inflammation. Accordingly, the present study was designed as a pilot investigation aimed at comparing serum miRNA-223-3p and IL-1β expression levels, along with VAS scores, between fibromyalgia patients and healthy individuals.

## 2. Results

### 2.1. Demographic Data

The demographic characteristics of the patient and control groups are summarized in Table 1. The study included a total of 36 participants, with an equal number of individuals in each group (*n* = 18). Although the proportion of women was higher than that of men in both the patient group (77.8%) and the control group (61.1%), no statistically significant difference was observed between the groups (*p* = 0.2777). The mean age was calculated as 46.2 years in the patient group and 48.5 years in the control group. The average height and weight of the patient group were 160.5 cm and 71.9 kg, respectively, whereas these values were 162.9 cm and 70.9 kg in the control group. No significant differences were detected between the groups with respect to these variables (*p* = 0.5023 and *p* = 0.5730).

When comparing mean body mass index (BMI), the patient group exhibited a mean BMI of 28.1, while the control group had a mean BMI of 26.0. Statistical analyses indicated that there were no significant differences between the groups in terms of age, sex, or BMI. Furthermore, when BMI was evaluated categorically (<25, 25–30, >30), no statistically significant differences were identified between the groups (*p* = 0.1527).

In the patient group, pain severity was assessed using the Visual Analog Scale (VAS). Based on this 0–10 scoring system, the mean VAS score was recorded as 8.1, indicating that participants in the patient group experienced a markedly high level of perceived pain.

### 2.2. miRNA-223-3p Expression Levels

**Figure 1:**
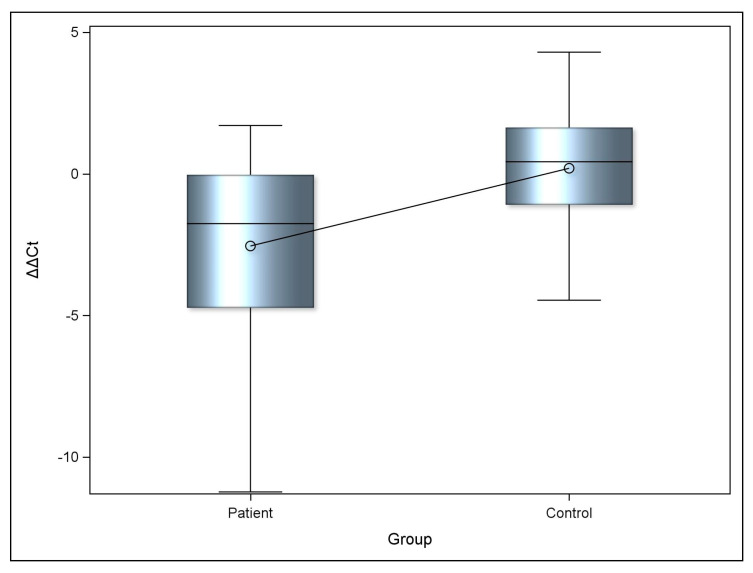
miRNA-223-3p expression levels between groups. MiRNA expression levels were also analyzed according to sex, and no statistically significant difference was detected between females and males (*p* = 0.463).

Descriptive statistics and comparative analyses of miRNA-223-3p levels in the patient and control groups are summarized in Table 2. Relative expression differences between the patient and control groups were evaluated using the ΔΔCT method [50]. As shown in the table, a statistically significant difference was observed between the two groups. Notably, serum miRNA-223-3p levels were markedly reduced in the FMS group compared with the control group, a finding that is also illustrated in Figure 1.

### 2.3. Evaluation of the Diagnostic Potential of miRNA-223-3p (ROC Analysis)

The diagnostic performance of miRNA-223-3p levels in distinguishing disease status was evaluated using Receiver Operating Characteristic (ROC) analysis. The optimal cut-off value was determined according to the Youden Index (Youden Index = Sensitivity + Specificity − 1), and the corresponding sensitivity and specificity values were calculated. Additionally, an empirical ROC curve based on this data was generated in SAS software using a nonparametric approach.

**Figure 2:**
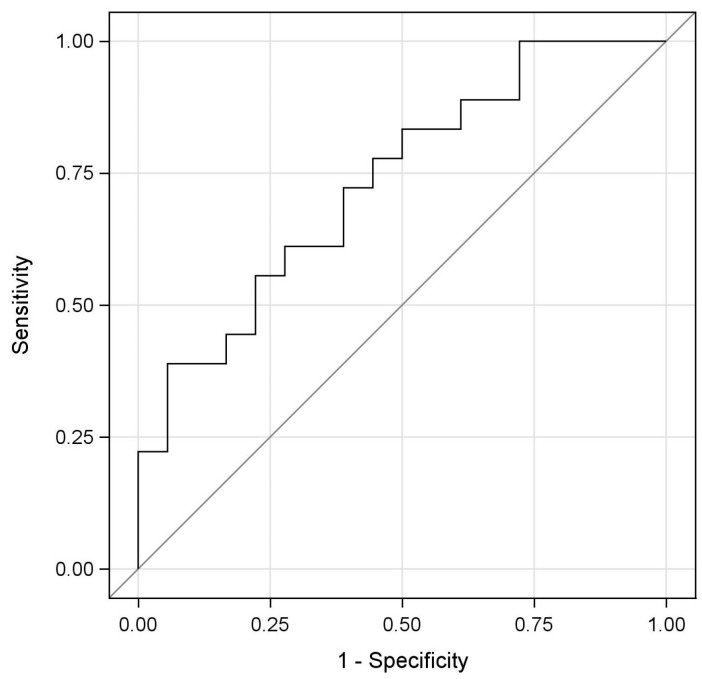
miRNA-223-3p ROC analysis diagram.

The results of the ROC analysis for miRNA-223-3p are presented in Table 3, and the corresponding ROC curve is illustrated in Figure 2. The analysis yielded an Area Under the Curve (AUC) value of 0.731, with a 95% confidence interval of 0.569–0.894 and a *p*-value of <0.05.

### 2.4. IL-1β Serum Values

In our study, the difference in serum IL-1β concentrations measured using the ELISA method was not statistically significant (*p* = 0.1135).

### 2.5. Correlation Between VAS, BMI, IL-1β and miRNA-223-3p Expression Levels

A correlation analysis was performed to evaluate the relationship between BMI, VAS score, miRNA-223-3p and IL-1β levels in the patient group, and the results are presented in Table 4. The table shows that there is a negative, moderate to high, and statistically significant correlation between VAS score and miRNA-223-3p levels (r = −0.6699, *p* = 0.0024) and no statistically significant correlation between miRNA-223-3p expression levels and BMI and IL-1β values.

## 3. Discussion

In the present study, circulating miRNA-223-3p expression levels were examined alongside VAS scores and IL-1β concentrations in individuals with fibromyalgia, revealing a significant association between miRNA-223-3p and pain severity. Fibromyalgia represents a multifactorial and clinically heterogeneous syndrome in which diagnostic precision remains suboptimal, primarily due to the reliance on subjective symptom assessments within current classification criteria [2,51]. Therefore, the lack of robust and objective biomarkers continues to hinder both early diagnosis and the development of targeted, mechanism-based therapeutic strategies

In this context, the regulatory involvement of microRNAs in pain-related processes—together with variable expression profiles in fibromyalgia and the negative correlation between miRNA-223 levels and pain intensity in lumbar radicular pain and complex regional pain syndrome—supports a modulatory role for this molecule in the pathophysiology of pain [21,52,53]. Overexpression of miRNA-223-3p has been reported to suppress cellular injury and inflammation across diverse inflammatory pain models, including postherpetic neuralgia, glioblastoma, and intervertebral disc herniation [28,54,55,56]. In murine studies, this upregulation also alleviated trigeminal neuropathic pain and was accompanied by reduced levels of pro-inflammatory cytokines [29]. Furthermore, the literature suggests disease-specific regulatory patterns for this microRNA across neurological disorders, and an association with fibromyalgia has likewise been documented [22,57]. In this context, the relationship between miRNA-223-3p expression levels and pain severity—also observed in our study—may contribute to more objective diagnostic approaches for FMS and support the potential for molecularly informed, personalized therapeutic strategies targeting underlying mechanisms [4,58,59].

Cerebrospinal fluid (CSF) and serum analyses in patients with FMS have demonstrated significantly reduced levels of miRNA-223-3p compared with healthy individuals. For instance, despite its putative role in suppressing oxidative stress, miRNA-223-3p has been reported to be markedly downregulated (6–13-fold) in FMS patients relative to controls [60]. This inadequate regulation may lead to insufficient control of oxidative stress, thereby contributing to the pathophysiology of the disease. Notably, miRNA-223-3p inhibition has been shown to exacerbate apoptosis and oxidative damage in Radiation-Induced Heart Disease (RIHD) mouse model [61]. Consistently, Cerda-Olmedo et al. reported a significant downregulation of miRNA-223-3p in FMS patients [59]. Also, in the CSF of women with FMS presenting with inflammatory pain, levels of this miRNA were found to be markedly lower than in healthy controls [5,62]. Our findings corroborate these observations, as serum miRNA-223-3p concentrations in FMS patients were significantly lower compared to healthy counterparts. The repeated association of miRNA-223-3p with FMS across different biofluid types suggests that this molecule may have potential relevance in the disease process; however, further studies are required before it may be considered a reliable biomarker candidate.

Notably, the differences in miRNA-223-3p expression observed here may not be solely driven by fibromyalgia-specific mechanisms and could, at least in part, reflect variations in inflammatory burden. Accordingly, IL-1β levels were additionally assessed, and the findings were interpreted in relation to miRNA-223-3p expression.

The evidence regarding IL-1β levels in fibromyalgia is highly inconsistent across literature. Several studies have reported no significant elevation in IL-1β levels and have found no statistically significant association between VAS scores and serum IL-1β in patients with FMS [63,64,65,66]. Some studies have even reported reduced concentrations [67]. In one investigation, levels were below the detection threshold, suggesting that this cytokine may be markedly low in certain individuals [39]. Conversely, some other studies have documented elevated IL-1β levels in fibromyalgia patients [68,69,70]. Consistent with prior studies reporting no significant differences in IL-1β levels, our findings indicated no statistically significant difference in IL-1β concentrations between the patient and control groups. Likewise, no statistically significant association was observed between serum IL-1β levels and VAS pain scores. However, these findings may have been influenced by insufficient statistical power due to the limited sample size; therefore, replication in larger cohorts, preferably in multicenter studies, is warranted for confirmation.

Finally, the obtained data were evaluated in conjunction with demographic characteristics and VAS pain scores. In a study conducted by Bjersing et al. using cerebrospinal fluid samples, no significant association was identified between Fibromyalgia Impact Questionnaire (FIQ) pain scores and miRNA-223-3p levels [5]. Methodological differences between studies may account for this discrepancy. Notably, the use of FIQ rather than VAS for pain assessment, as well as the very limited sample size in that study (*n* = 10), are likely contributors to the divergent findings. In contrast, our study demonstrated a negative correlation between VAS scores and serum miRNA-223-3p levels, indicating that higher pain severity was associated with lower expression of this molecule. This negative relationship suggests that miRNA-223-3p may play a regulatory role in pain mechanisms. Furthermore, existing evidence supports its potential anti-inflammatory and neuroprotective effects and indicates that reduced expression levels of miRNA-223-3p may be linked to increased pain severity [26,29,52].

In this study, the diagnostic performance of miRNA-223-3p, as determined through ROC curve analysis, was characterized by high specificity and moderate sensitivity. This profile suggests that miRNA-223-3p may function as a robust confirmatory biomarker capable of supporting the presence of disease, while simultaneously exhibiting limitations in identifying all affected individuals. The wide confidence interval surrounding the AUC value further indicates potential variability in diagnostic precision, likely attributable to sample size constraints.

Of particular significance, a key strength and novelty of the present study is that it constitutes the first investigation to simultaneously assess miRNA-223-3p expression, IL-1β concentrations, and pain severity measured by VAS in individuals with FMS. This unique contribution addresses a notable gap in the literature and offers an important initial framework for understanding the potential biomarker role of miRNA-223-3p in fibromyalgia pathogenesis. Moreover, the observed associations in this study provide preliminary evidence that miRNA-223-3p may also be relevant to other chronic pain syndromes, consistent with its broader involvement in immune regulation and neuroinflammatory signaling.

While this study has strengths, it also has limitations. Although the sample size was deemed statistically adequate based on G*Power 3.1 (version 3.1.9.7), the relatively small number of participants may limit the generalizability of the findings. In addition, the cross-sectional design and reliance on measurements obtained at a single time restrict causal inference regarding the relationship between miRNA-223-3p levels and pain severity and preclude assessment of temporal fluctuations in miRNA expression. Additionally, hormonal status, dietary factors, and oxidative stress markers were not evaluated as part of this study.

Furthermore, the use of circulating miRNAs as diagnostic biomarkers is subject to both biological and technical constraints that apply broadly to miRNA-based studies. Circulating miRNA levels can be influenced by factors such as sampling time, hemolysis, and pre-analytical/analytical laboratory procedures; moreover, the tissue of origin is often difficult to ascertain, thereby complicating biological interpretation.

As the first study to jointly evaluate miRNA-223-3p, IL-1β, and VAS scores, our findings should be regarded as hypothesis-generating. In this context, although our sex-based statistical analyses indicated that gender did not have a measurable effect on miRNA-223-3p expression in this cohort, we plan to conduct studies with larger sample sizes, including BMI- and sex-stratified subgroup analyses, and to employ multivariable models incorporating inflammatory and oxidative stress biomarkers, as well as hormonal status and dietary factors. Such approaches will be critical for more clearly elucidating the underlying mechanisms.

## 4. Materials and Methods

### 4.1. Study Design and Sampling

This study was designed as a single-center, cross-sectional, case–control, pilot investigation. The sample size was determined through power analysis to ensure adequate statistical power for the study.

### 4.2. Case and Control Groups

Between June 2024 and June 2025, individuals diagnosed with FMS according to the ACR criteria, who were presented to the Department of Physical Medicine and Rehabilitation at Akdeniz University Hospital and met the inclusion criteria, were consecutively recruited. Patient data were obtained from medical records.

Inclusion criteria:

- Age over 18 years.
- Voluntary participation with a confirmed diagnosis of FMS.

Exclusion criteria:

- Infectious or inflammatory disease.
- Chronic diseases.
- Neurological or Psychiatric disorder.
- Active use of analgesic, antidepressant, or anti-inflammatory medications.
- Chronic fatigue syndrome or other pain syndromes.
- Pregnancy.
- Antibiotic use within the last three months.

The control group consisted of completely healthy volunteers, aged over 18 years, matching the cases by age and gender. Controls were consecutively recruited from the same social environment as the cases and were required to meet the same exclusion criteria.

### 4.3. Data Collection Methods

The detailed demographic characteristics of all participants, including age, sex, height, weight, and body mass index (BMI), were systematically recorded. To assess pain severity, the Visual Analog Scale (VAS) was administered to all participants. To minimize circadian rhythm-related biological variability, blood samples were collected from all participants between 09:00 and 10:00 a.m. using a standardized venous sampling protocol. Within the scope of the study, peripheral blood samples were collected into plain vacuum gel tubes and centrifuged at 3500× *g* for 5 min (refrigerated centrifuge) to obtain serum. The resulting serum samples were transferred into sterile tubes and stored at −80 °C until miRNA analyses were performed.

#### Assessment of Pain Severity

In this study, VAS was employed to assess pain severity in patients with FMS. The VAS consists of a 10 cm straight line without markings or numbers; the left end represents “no pain” (0 cm), and the right end represents “unbearable pain” (10 cm). Participants were asked to mark the point on the line that they believed most accurately reflected the intensity of their pain. The distance from the marked point to the 0 cm position was recorded as the pain severity score. This score ranges from 0 to 10, with greater distances indicating higher pain intensity. In this way, each individual’s pain experience was evaluated using an objective measurement scale.

### 4.4. miRNA-223-3p Expression Analysis

miRNA Isolation: On the day of the experiment, serum samples were thawed and centrifuged at 1400× *g* at +4 °C for 20 min. MiRNA isolation was then performed from the supernatant using the miRNeasy Serum/Plasma Kit (Cat. No./ID: 217184, Qiagen, Hilden, Germany) according to the manufacturer’s protocol. The purity and concentration of the isolated miRNAs were assessed using a NanoDrop 2000 spectrophotometer (Thermo Fisher Scientific, Waltham, MA, USA).

cDNA Synthesis: Complementary DNA (cDNA) synthesis was performed using the miRCURY LNA RT Kit (Cat. No./ID: 339340, Qiagen, Hilden, Germany). During the cDNA synthesis process, the isolated miRNA samples underwent reverse transcription.

Determination of miRNA Levels Using Fluorometry: The concentration of the transcribed miRNAs was measured using the Qubit 3.0 Fluorometer (Thermo Fisher Scientific, Waltham, MA, USA) by following the standard protocol of the Qubit^TM^ miRNA Assay Kit (Invitrogen/Thermo Fisher Scientific, Waltham, MA, USA). Appropriate dilutions were made after concentration measurements.

miRNA Expression Analysis: Following quantification of the sample concentrations, the expression levels of microRNA-223-3p (miRCURY 223-3p, Kat. No./ID: 205986-1, Qiagen, Hilden, Germany) were measured using the Rotor-Gene Q Real-Time PCR System (Qiagen, Hilden, Germany; software version: 2.3.5) with the miRCURY LNA SYBR Green PCR Kit (Cat. No./ID: 339346, Qiagen, Germantown, MD, USA). RNU6 (Lot: 20800469-1, Qiagen) was used as the endogenous reference gene for normalization of ΔCT values. CT values obtained from the instrument were used to calculate ΔCT for each sample, and relative expression differences between the patient and control groups were evaluated using the ΔΔCT method.

### 4.5. Measurement of Serum IL-1β Levels by ELISA

Serum IL-1β concentrations were quantified using a commercially available sandwich enzyme-linked immunosorbent assay (ELISA) kit (Human IL-1β ELISA Kit, ABT1074Hu, Ankara, Türkiye) according to the manufacturer’s instructions. ELISA kits were stored at +4 °C under conditions specified by the manufacturer. Prior to the assay, serum samples and all reagents were equilibrated to room temperature (18–25 °C), and reagent preparation was performed as recommended. The assay is based on a microtiter plate pre-coated with a monoclonal antibody specific to human IL-1β. Standards and serum samples were added to the wells to allow antigen binding to the immobilized capture antibody. After washing to remove unbound material, a biotin-conjugated detection antibody specific to IL-1β was added, followed by incubation with an avidin–horseradish peroxidase (HRP) conjugate. Excess conjugate was removed by additional washing steps.

A chromogenic substrate solution was then added to each well, resulting in the development of a blue color in proportion to the amount of bound IL-1β. The reaction was stopped by the addition of stop solution, which converted the color from blue to yellow. Optical density (OD) was measured at 450 nm using a SYNERGY/HTX Multi-Mode microplate reader (BioTek Instruments, Winooski, VT, USA). Serum IL-1β concentrations (pg/mL) were calculated using the standard concentration–absorbance curve. All samples were assayed in duplicate to ensure analytical reliability.

### 4.6. Statistical Analyses

Statistical analyses of the data obtained in this study were performed using the SAS 9.4 (SAS Institute Inc., Cary, NC, USA) software package. For quantitative variables measured in the study, descriptive statistics were presented as mean and standard deviation, while qualitative variables expressed in numbers were summarized as frequency (n) and percentage (%).

The normality of the distribution of the variables was first assessed using the Kolmogorov–Smirnov test. In addition, skewness values were examined. Based on these tests, since the skewness coefficients for all variables were not within the range of +3 to −3, the data were determined not to follow a normal distribution. Accordingly, non-parametric tests were preferred in statistical analyses [71]. The Mann–Whitney U test was used to compare two independent groups. In addition, correlation analyses were performed to evaluate the relationships among BMI, VAS scores, miRNA-223-3p expression, and IL-1β levels.

The data were further analyzed in terms of sensitivity and specificity values derived from receiver operating characteristic (ROC) curves. ROC curves were constructed to determine optimal cut-off values for the index tests, and areas under the curve (AUC) were calculated. Throughout the study, statistical significance was set at *p* < 0.05.

## 5. Conclusions

This study yields findings consistent with existing evidence implicating miRNA-223-3p in the pathophysiology of fibromyalgia. The observed inverse correlation between miRNA-223-3p expression and pain severity indicates that this miRNA may contribute to the modulation of pain-related mechanisms in FMS. Given the inherent biological variability of circulating miRNAs, further research incorporating larger cohorts and multi-biomarker analytical frameworks is warranted to clarify the diagnostic and therapeutic relevance of miRNA-223-3p in fibromyalgia.

## Figures and Tables

**Table 1:** Demographic characteristics of the patient and control groups.

	Patient	Control	*p*-Value
Gender, *n* (%)			0.2777 ^1^
Male	4 (22.2%)	7 (38.9%)	
Female	14 (77.8%)	11 (61.1%)	
Age			0.6806 ^2^
Mean (SD)	46.2 (11.64)	48.5 (12.12)	
Median (Range)	45.5 (23.0, 73.0)	47.0 (27.0, 73.0)	
BMI			0.1455 ^2^
Mean (SD)	28.1 (5.02)	26.0 (4.16)	
Median (Range)	26.8 (19.5, 37.8)	24.6(21.3, 40.0)	
VAS score			
Mean (SD)	8.1 (1.43)		
Median (Range)	8.0 (5.0, 10.0)		

^1^ Chi-Square *p*-value; ^2^ Kruskal–Wallis *p*-value.

**Table 2:** Comparison of miRNA-223-3p expression levels between the patient and control groups.

ΔΔCT	Patient	Control	*p*-Value
Mean (SD)	−2.5 (3.3)	0.2 (2.52)	0.0176 *
Median (Range)	−1.8 (−11.2, 1.7)	0.4 (−4.5, 4.3)	

* Mann Whitney U *p*-value.

**Table 3:** miRNA-223-3p ROC analysis results.

	AUC	SE	95%CI		*p*	Cut Off	Sensitivity	Specificity
∆∆Ct	0.731	0.083	0.569	0.894	0.005	0.96	0.389	0.944

**Table 4:** Correlation analysis.

	BMI	VAS	miRNA-223-3p	IL-1β
BMI	1.0000			
VAS	0.3343	1.0000		
	0.1751			
miRNA-223-3p	−0.1915	−0.6699	1.0000	
	0.2630	0.0024		
IL-1β	0.0315	−0.0485	0.0192	1.0000
	0.9012	0.8489	0.9394	

## Data Availability

The data presented in this study are available on request from the corresponding author. The data are not publicly available due to privacy or ethical restrictions.

## Abbreviations

The following abbreviations are used in this manuscript:

FMS	Fibromyalgia Syndrome
miRNA	microRNA
VAS	Visual Analog Scale
ROS	Reactive Oxygen Species
NLRP3	NOD-like receptor protein 3
FOXO1	Forkhead box protein O1
FBXW7	F-box and WD repeat domain-containing 7
SORBS1	Sorbin and SH3 domain-containing protein 1.
iNOS	Inducible Nitric Oxide Synthase
NF-Κb	Nuclear Factor kappa-light-chain-enhancer of activated B cells
IL-1β	Interleukin-1 beta
IL-6	Interleukin-6
TNF-α	Tumor Necrosis Factor alpha
BMI	Body Mass Index
CSF	Cerebrospinal Fluid
ROC	Receiver Operating Characteristics
FIQ	Fibromyalgia Impact Questionnaire
MNK2	MAP kinase-interacting serine/threonine-protein kinase 2
MAPK	Mitogen-Activated Protein Kinase
